# Recent advances in the understanding and management of atrial fibrillation: a focus on stroke prevention

**DOI:** 10.12688/f1000research.10176.1

**Published:** 2016-12-20

**Authors:** Farhan Shahid, Eduard Shantsila, Gregory Y. H. Lip

**Affiliations:** 1 University of Birmingham Institute of Cardiovascular Sciences, Birmingham, UK; 2 Aalborg Thrombosis Research Unit, Department of Clinical Medicine, Aalborg University, Aalborg, Denmark

**Keywords:** Atrial Fibrillation, screening, stroke, CHA2DS2VASc, HAS BLED, anti-coagulation, warfarin, non-vitamin k anti coagulants

## Abstract

Atrial fibrillation (AF) is associated with an increased risk of stroke compared with the general population. It is anticipated that by 2030 an estimated 14–17 million patients will be diagnosed with this most prevalent arrhythmia within the European Union. AF-related stroke confers a higher mortality and morbidity risk, and thus early detection and assessment for the initiation of effective stroke prevention with oral anticoagulation (OAC) is crucial. Recent guidelines point to the use of non-vitamin K antagonist OACs (NOACs) where appropriate in stroke prevention of patients with non-valvular AF. At present, there are four NOACS available, with no direct head-to-head comparisons to suggest the superiority of one drug over another.

Simple and practical risk assessment tools have evolved over the years to facilitate stroke and bleeding risk assessment in busy clinics and wards to aid decision-making. At present, the CHA _2_DS _2_VASc (congestive heart failure, hypertension, age 65–74/>75, diabetes mellitus, stroke/transient ischemic attack/thromboembolism, vascular disease, female sex) score is recommended by many international guidelines as a simple and practical method of assessing stroke risk in such patients. Alongside this, use of the HAS BLED (hypertension systolic blood pressure >160 mmHg, abnormal liver/renal function [with creatinine ≥200 μmol/L], stroke, bleeding history or predisposition, labile international normalized ratio [range <60% of the time], elderly [>65], concomitant drugs/alcohol) score aims to identify patients at high risk of bleeding for more regular review and follow-up and draws attention to potentially reversible bleeding risk factors.

The aim of this review article is to provide an overview of recent advances in the understanding and management of AF with a focus on stroke prevention.

## Introduction

Atrial fibrillation (AF) is associated with a five-fold increase in the risk of stroke, and AF-related stroke patients have a higher mortality and greater morbidity than patients with non-AF-related stroke ^1^. It is anticipated that by 2030 an estimated 14–17 million patients will be diagnosed with this most prevalent arrhythmia within the European Union.

Over recent years, the need for early detection and use of appropriate thromboprophylaxis have proved to be central in the prevention of AF-related stroke, which in itself carries a higher morbidity and mortality than non-AF-related stroke ^2^. The use of oral anticoagulation (OAC), whether with the vitamin K antagonists (VKAs, e.g. warfarin) or, more recently, the non-VKA OACs (NOACs), results in a marked reduction in stroke and all-cause mortality ^3, 4^. Many guidelines now emphasize that the default should be to offer thromboprophylaxis to all patients with AF, unless “truly low risk” is evident such that OAC confers no advantage ^5^. Aspirin has been proven to offer little net clinical benefit and is not recommended for stroke prevention in AF ^6^.

Various systemic reviews have highlighted the common risk factors associated with AF-related stroke ^1, 7^. There are a number of independent “stroke risk factors”, but each may not necessarily contribute equally to stroke risk in AF. To aid in the practical evaluation of stroke risk in AF, various risk stratification schemes have been proposed to aid decision-making regarding thromboprophylaxis ^8^. Such schemes are based on risk factors derived from the non-VKA arms of the historical clinical trial cohorts, large observational studies, and consensus opinion, and the resulting schemas vary greatly in their complexity and number of risk factors ^9^. Even one stroke risk factor confers excess risk of stroke and mortality. In essence, patients with risk factors should be offered OAC unless contraindicated, given the positive net clinical benefit for treating such patients ^6, 10, 11^.

The aim of this review article is to provide an overview of the recent advances in the diagnosis and management of patients with AF with a focus on stroke prevention.

## Pathophysiology of atrial fibrillation and its complications: a brief overview

External stressors such as hypertension, diabetes mellitus, and AF itself can stimulate a process of atrial remodeling and subsequent fibrosis, which acts as a substrate for AF (along with other cardiac arrhythmias) ^12^. The structural remodeling that takes place leads to an alteration in the electrical conduction pathway in the atrium, leading to a low threshold re-entry circuit and propagation of arrhythmias ^13^. AF itself takes place after the process of cardiac remodeling and fibrosis. Thus, treatment aimed at minimizing this adverse remodeling pathway should be initiated at the earliest opportunity ^14^.

The rhythm of AF itself along with the structural remodeling that takes place predisposes the atrial myocardium to a prothrombotic state (especially within the left atrial appendage) ^15^. Furthermore, short episodes of AF can cause myocardial damage within the atrium, which in turn stimulates the release of prothrombotic factors onto the endothelial surface, leading to the aggregation of platelets. This, in part, explains why even short episodes of AF can confer long-term stroke risk ^16, 17^.

The mechanisms that cause AF are heterogeneous. For example, in patients with structural heart disease, there is a prolonged atrial refractory period that acts as the substrate to AF, whereas patients who develop AF in the absence of ischemic heart disease often have a shortening of the atrial refractory period due to the downregulation of inward calcium channels and the upregulation of potassium inward currents ^18, 19^. This alteration in calcium handling by the atrial myocardium in line with atrial remodeling appears to be the most plausible explanation of how changes in autonomic tone can initiate AF ^20^.

## Screening for atrial fibrillation

The adverse outcomes associated with AF are preventable by the appropriate and timely introduction of medical therapy. Given the fact that AF-related stroke carries with it a poorer outcome than does non-AF-related stroke, the appropriate use of OACs provides a means by which the detrimental thromboembolic effects of AF can be avoided.

In an ideal setting, AF would be negated by the introduction of effective primary preventative therapies, with the next best option being the early initiation of treatment if and when AF is detected. However, with 30% of AF being found in asymptomatic patients, how best to detect this arrhythmia is of some growing concern ^21^. A proportion of patients are fortunate enough to have AF detected by chance, often because of routine medical examinations for other reasons.

The absence of symptoms does not remove or reduce the risk of associated stroke, with this cohort of patients often found to have a higher CHA _2_DS _2_VASc (congestive heart failure, hypertension, age 65–74/>75, diabetes mellitus, stroke/transient ischemic attack [TIA]/thromboembolism, vascular disease, female sex) score than symptomatic patients ^22^. Unfortunately, for the vast majority of patients with asymptomatic AF, the first opportunity to detect this arrhythmia is in the context of an acute stroke ^2^. One in five ischemic strokes are attributable to AF, of which greater than 20% represent AF diagnosed after the stroke event ^23^. Without question, such events could have been avoided with earlier detection and initiation of OAC.

The suggestion of widespread screening for AF is not new, with previous studies within community healthcare practices and meta-analyses showing a clear benefit ^24^. However, the optimal method by which to go about detecting asymptomatic AF is unclear.

The cryptogenic stroke and underlying AF (CRYSTAL AF) study was designed to evaluate whether continuous cardiac monitoring in the way of an implantable cardiac monitor was superior to detecting AF versus “conventional follow-up” in patients with a cryptogenic stroke or TIA ^25^. During the 3-year study period, 447 patients were enrolled into this study. Of the 221 patients receiving an implantable cardiac monitor, 8.9% had AF detected at 6 months versus only 1.4% in the control group. Importantly, the benefit of prolonged monitoring was maintained at 12-month follow-up, with a 12.4% AF detection rate in the implantable cardiac monitoring group. Most poignantly, ischemic stroke or TIA occurred in 11 patients with the intracardiac monitor versus 18 patients in the control group. This favorable outlook in the intracardiac monitor group corresponded to a higher use of OACs at the 6-month interval (10.1% versus only 4.6% in the control group).

The EMBRACE trial was another randomized controlled trial aimed at quantifying the benefit of longer monitoring periods for patients with potential AF in the context of secondary prevention of stroke ^26^. Of the 572 patients enrolled, 16.2% of patients had at least 30 seconds of AF detected over 90 days of monitoring compared to only 3.2% in those who underwent 24-hour monitoring. This correlated with an absolute difference of OAC uptake of 7.5% in favor of prolonged monitoring.

Recently, the SEARCH AF study analyzed the feasibility and cost-effectiveness of opportunistic, community-based screening in Australia in patients aged over 65 ^22^. A structured screening method including a brief history and pulse palpation, and a handheld phone-based ECG recording was taken. A total of 1000 pharmacy customers were screened with newly identified AF in 1.5% of the cohort. The sensitivity and specificity for this automated iECG algorithm was 98.5% and 91.4%, respectively. A cost-effective analysis showed that most benefit was observed in relation to quality-adjusted life years in those patients in whom anticoagulation adherence was optimal.

With the fruition of large randomized studies showing a clear benefit for prolonged monitoring in patients at risk of AF along with a cost benefit, guidelines will no doubt begin to incorporate a more structured approach for the detection of asymptomatic AF. At present, pulse palpation and ECG rhythm strip are recommended for primary prevention and short-term monitoring of at least 72 hours in those patients having suffered a TIA or ischemic stroke ^5^.

## How much atrial fibrillation is significant?

For a long time, evidence-based guidelines have questioned whether “AF burden” is relevant to stroke risk. As such, current guidelines do not distinguish between types of AF with regard to thromboprophylaxis, as observational data suggest that stroke risk is similar regardless of AF subtype in the presence of stroke risk factors ^27, 28^. The European Atrial Fibrillation Trial (EAFT) with a follow-up of 594 patient-years found AF duration >1 year was an independent risk factor for secondary stroke ^29^.

One meta-analysis examined stroke rates in 134,847 patients with paroxysmal AF (PAF) versus permanent AF off anticoagulation and reported an odds ratio (OR) of 0.75 (95% confidence interval [CI] 0.61–0.93) in favor of less stroke risk in patients with PAF ^30^. In anticoagulated patients, the OR also favored patients with PAF (OR 0.77, 95% CI 0.68–0.88). This was confirmed in a further systematic review and meta-analysis ^31^.

Furthermore, analysis of data from “The AF Clopidogrel Trial with Irbesartan for prevention of vascular events” (ACTIVE-A) and “Apixaban Versus Acetylsalicylic Acid to Prevent Stroke in AF Patients Who Have Failed or Are Unsuitable for VKA Treatment” (AVERROES) trials also pointed to the idea that a pattern of AF was a strong independent predictor of stroke risk, second only to previous TIA or stroke ^32^. In the 6,563 patients included in this analysis, permanent AF had an annual stroke risk of 4.2% compared to 2.1% with PAF and 3.0% with persistent AF. Hazard ratios of 1.83 for permanent AF versus PAF and 1.44 for persistent AF versus PAF were found, respectively. A subanalysis of the “Rivaroxaban Once daily oral direct factor Xa inhibition compared with VKA for prevention of stroke and Embolism Trial in AF” (ROCKET AF) also found that anticoagulated patients with persistent AF (11,548 patients) were at higher risk of stroke versus those with PAF (2,514 patients) ^33^. Patients with persistent AF had higher rates of stroke and all-cause mortality (adjusted rates for stroke 2.18 versus 1.73 events/100 patient-years, p=0.048).

Despite trials showing that the pattern of AF has an impact on stroke risk, there appears to be marked heterogeneity amongst the respective trials, making comparisons difficult. Furthermore, the fluctuations in OAC use between trials makes conclusive links between patterns of AF and stroke risk difficult to extrapolate. Within types of AF, there can be marked heterogeneity. In PAF, for example, those with one paroxysm once a year are labeled as PAF, as would a patient with paroxysms of AF 364 days per year.

Therefore, at present, patients diagnosed with AF, regardless of type or duration, require assessment for stroke and bleeding risk using guideline recommendations ^5, 34^.

## Anticoagulation in patients with atrial fibrillation

Stroke prevention in patients with non-valvular AF requires careful consideration of the risk versus benefit of starting OAC therapy. Stroke and bleeding risk factors in patients with AF are not homogeneous, and risk stratification schemes such as the CHA _2_DS _2_VASc and HAS BLED (hypertension systolic blood pressure >160 mmHg, abnormal liver/renal function [with creatinine ≥200 μmol/L], stroke, bleeding history or predisposition, labile international normalized ratio [INR] [eg. Time in Therapeutic Range <60%], elderly ^>65^, concomitant drugs/alcohol) scores are well validated and provide a simple and quick yet concise method of assessing a patient’s suitability for anticoagulation without the necessity of complex composite scores or multiple biomarkers ^8, 35^.

For more than 50 years, the VKAs, e.g. warfarin, have been the mainstay of anticoagulation in patients with non-valvular AF, significantly reducing stroke and mortality ^36^. Aspirin monotherapy, on the other hand, is ineffective for stroke prevention and indeed unsafe ^6^.

In 2009 came the introduction of the NOACs, which revolutionized the management of stroke prevention in non-valvular AF. Initially referred to as new or novel OACs, or sometimes direct OACs (DOACs), the NOAC acronym has been retained to refer to non-VKA OACs ^37, 38^. The four major drugs (dabigatran, apixaban, rivaroxaban, and edoxaban) compare favorably with warfarin, showing at least non-inferiority in regards to stroke prevention, with a superior safety profile with regard to major bleeding ^39– 42^. Recent data from ancillary analyses of the major trials show that patients taking NOACs are at 30–50% lower risk of major bleeding than with warfarin ^43– 45^. As yet, no head-to-head trials amongst the NOACs have been undertaken.

### Dabigatran

Dabigatran is an oral direct thrombin inhibitor, binding to the active catalytic site of thrombin in a reversible manner. This action blocks the conversion of fibrinogen to fibrin. Dabigatran is available as a non-active pro-drug that is converted to its active form *in vivo* (gut mucosa, liver, and plasma). Renal elimination is the dominant pathway of excretion for this anticoagulant (up to 80%).

The efficacy and safety of dabigatran was first evaluated in a large randomized controlled study in 2009. The RE-LY study compared dabigatran (150 and 110 mg bis die [b.d.]) to dose-adjusted warfarin. Efficacy analysis showed non-inferiority of the 110 mg b.d. dose (1.54%/year) and superiority of the 150 mg b.d. dose (1.11%/year) compared to warfarin (1.71%/year) for the prevention of stroke and systemic embolism ^39, 46^. Both dosing regimens were associated with lower risk of intracranial hemorrhage (ICH) compared to warfarin ^39, 46^. This was consistent across a range of stroke risk strata ^47^. Of note, however, was the higher gastrointestinal bleeding risk with dabigatran 150 mg b.d. (1.51% versus 1.02%/year for warfarin) ^39^. Dabigatran 150 mg b.d. was associated with a lower cardiovascular mortality ^39, 46^, which has been confirmed in a recent meta-analysis of observational data ^48^. Since the RE-LY trial, subsequent “real world” evidence has provided further supportive evidence for the superiority of dabigatran over warfarin ^49– 51^.

### Apixaban

Apixaban is a factor Xa inhibitor that is approved for patients with non-valvular AF in need of stroke prevention. Major trials excluded patients with a creatinine level of 2.5 mm/dL or a creatinine clearance of <25 mL/min/1.73 m ^2^. A dose reduction is available from 5 mg b.d. to 2.5 mg b.d. for patients who have two of the following factors: age ≥80 years, weight <60 kg, or serum creatinine >1.5 mg/dL ^52, 53^.

In 2011, the AVERROES trial showed a clear benefit of apixaban over aspirin ^54, 55^. There were no significant differences in the risk of major bleeding or ICH between apixaban and aspirin. In 2012, the ARISTOTLE (apixaban for the reduction in stroke and other thrombotic events in atrial fibrillation) trial compared apixaban to warfarin in 18,201 patients ^41^ and found apixaban to be superior to warfarin for the primary outcome of stroke and systemic embolism (1.27% versus 1.6%, respectively). Apixaban was also associated with a significantly lower incidence of major bleeding, ICH, and mortality ^56^. In recent observational data, apixaban has been shown to provide greater treatment persistence versus warfarin in AF patients, which in itself could lead to fewer cardioembolic events and stroke burden ^57^.

### Rivaroxaban

The ROCKET AF trial enrolled 14,262 patients at moderate to high risk of stroke to either warfarin (target INR 2–3 or rivaroxaban 20 mg, with a dose reduction to 15 mg in those with creatinine clearance of 30–49 mL/min) ^40^. Rivaroxaban was non-inferior to warfarin for the composite end point of stroke and systemic embolism, with similar rates of major bleeding and ICH, but rivaroxaban had a higher incidence of gastrointestinal bleeding compared to warfarin. Prospective, non-interventional registries have provided reassuring data for rivaroxaban compared to VKAs, along with better treatment compliance ^58, 59^.

### Edoxaban

Like apixaban and rivaroxaban, edoxaban is a selective factor Xa inhibitor and was tested in the phase III ENGAGE AF TIMI-48 trial, which enrolled 21,105 patients to the high-dose edoxaban strategy arm, the low-dose strategy arm, or warfarin ^42^. The high-dose edoxaban arm was not inferior to warfarin for the primary endpoint of stroke and systemic embolism, with a significant reduction in major bleeding and ICH, although there were more gastrointestinal bleeds with edoxaban 60 mg versus warfarin. Efficacy appeared to diminish in patients with a high creatinine clearance, with edoxaban 60 mg once daily having a trend towards higher strokes with creatinine clearance of ≥95 mL/min, leading to a US Food and Drug Administration (FDA) black box for use in such patients. “Real world data” for edoxaban are limited, although indirect comparisons of edoxaban to anti-platelet therapies or placebo have been published ^60^.

### Reversal agents

There remain concerns regarding the bleeding risk with the NOACs and – until recently – the lack of a specific antidote ^61^. With the introduction of idarucizumab (a fully humanized antibody fragment) recently licensed for use in patients taking dabigatran, such concerns may be unwarranted ^62– 64^. In addition, andexanet alfa (a truncated form of enzymatically inactive factor Xa which binds factor Xa inhibitors and reverses their anticoagulant effects) was investigated for the reversal of oral factor Xa inhibitors ^65^. Also under development is ciraparantag ^66^, which is at an earlier stage of development as a universal reversal agent for all NOACs.

### Practical issues

As part of the initiation of NOAC therapy, the involvement of patient education is of central importance ^67, 68^. The patient must be made aware that missing a dose of NOAC potentially removes the stroke protection due to their relatively short half-life versus that of VKAs. Guidelines also emphasize the need for patient education and involvement in decision-making when deciding on the most appropriate anticoagulation ^5^. Thus, NOACs provide a better, safer, and more convenient anticoagulation option with a greater net clinical benefit ^69^. Accordingly, NOACs are now a well-established option (in addition to warfarin) for the prevention of thromboembolic events in non-valvular AF and venous thromboembolism and are given preference over warfarin in many updated clinical guidelines on the management of AF ^5, 34, 70^.

## How do clinical trial results compare with “real world data”?

Clinical trial data are not always reproducible in everyday clinical practice. Reassuringly, NOACs have continued to show a net clinical benefit when introduced in “real world” clinical settings, with the real world observational evidence being complementary and supportive of the trial results.

Dabigatran has been licensed and available the longest, hence many comparisons to warfarin in real world studies are with this direct thrombin inhibitor ^71^. The real world results for dabigatran have largely echoed the clinical trial findings ^72^. In a large US database compromising 12,793 patients with a mean age of 74 years, dabigatran was superior to warfarin with regard to stroke prevention (adjusted hazard ratio 0.73, 95% CI 0.55–0.97) with a lower incidence of major bleeding (adjusted hazard ratio for intracranial bleeding 0.49 [0.3–0.79]) ^73^. Other registry data have shown similar findings ^49, 74^. A recent meta-analysis of these observational data (with over 20 studies, totaling 711,298 patients) found a lower risk of ischemic stroke compared to warfarin (hazard ratio 0.86, CI 0.74–0.99) with a lower incidence of intracranial bleeding (0.45, CI 0.38–0.52) but higher risk of gastrointestinal bleeding (1.13, CI 1.00–1.28) ^51^.

Real world data regarding rivaroxaban and apixaban have recently also gathered pace. The XANTUS observational study was a prospective, observational study of patients treated with rivaroxaban for stroke prevention in AF. A total of 6,784 patients were initiated on rivaroxaban across 311 centers in Europe, Israel, and Canada. Rates of stroke were found to be low in this cohort of patients, with 43 patients suffering a stroke and 43 a major bleed (0.7 events per 100 patient-years and 2.1 events per 100 patient-years, respectively) ^75^. More recently, Coleman *et al*. compared data for AF patients newly started on rivaroxaban, apixaban, or warfarin ^76^. When compared to warfarin, rivaroxaban was associated with a reduction in ICH (0.49% versus 0.96% per year, hazard ratio 0.53, CI 0.35–0.79), with a non-significant reduction in ischemic stroke (0.54% versus 0.83% per year, hazard ratio 0.71, CI 0.47–1.07).

The current industry-funded GARFIELD AF registry aims to recruit between 55,000 and 60,000 patients with AF, analyzing trends of anticoagulant use in patients with AF. In the fourth cohort of GARFIELD AF, more than 70% of AF patients are anticoagulated, with a growing proportion being initiated on NOAC therapy over warfarin (37%), and OAC use was associated with a 35% lower risk of death ^77^. Other registry data have since been published for comparative effectiveness and safety data for dabigatran, rivaroxaban, apixaban, and warfarin ^50, 78, 79^.

## What does the future hold for non-vitamin K antagonist oral anticoagulation and stroke prevention?

With the superior net clinical benefit of NOACs over VKAs, the opportunity to offer anticoagulation to a wider spectrum of AF patients is clearly evident. Previously, with the use of warfarin, the inconvenience of multiple blood tests for INR monitoring and food/drug interactions meant not all eligible patients would accept or be offered OAC for stroke prophylaxis. With the need for minimal monitoring in regards to blood tests and a reduction in major bleeding risk, older and arguably frailer populations who have AF will now have the benefit of stroke prophylaxis without increasing their major bleeding risk to unacceptable levels. At present, patients with mechanical prosthetic heart valves are not eligible for NOAC therapy.

### What do we do for patients not suitable for OAC?

In a minority of cases, OAC may be absolutely contraindicated despite patients being at high risk of AF-related stroke. Aspirin is no longer recommended for stroke prophylaxis in patients with AF owing to its inferior efficacy in stroke prophylaxis along with an unacceptable heightened bleeding risk ^6^. After careful consideration by a multidisciplinary team and discussion with the patient, alternative interventions need to be sought.

Recent trial evidence has focused on the feasibility of left atrial appendage (LAA) closure devices. The PROTECT-AF trial compared the LAA device Watchman to warfarin in a randomized multi-center study of 707 patients, where patients were assigned in a 2:1 fashion to either LAA closure device with discontinuation of warfarin or warfarin with an INR target of 2–3 ^80^. Primary end point data (stroke, cardiovascular death, and systemic embolization) showed non-inferiority of the Watchman device over warfarin (event rate 3 per 100 patient-years in the intervention group versus 4.9 per 100 patient-years in the warfarin-treated cohort). Primary safety events (major bleeding, pericardial effusion, and device embolization) were more frequent in the intervention group than in the control group (7.4% per 100 patient-years versus 4.4% per 100 patients-years). Of note, 15% of patients in this trial remained on warfarin despite being in the interventional arm. At 3.8-year follow-up, long-term data from the PROTECT-AF trial appear to be consistent with initial analysis showing non-inferiority of Watchman to warfarin. The PREVAIL trial compared Watchman to warfarin in 407 high-risk patients (CHA _2_DS _2_VASc = 3.8) ^81^. The primary efficacy end points of stroke (hemorrhagic and ischemic), systemic embolization, and cardiovascular/unexplained death were similar (6.4% versus 6.3%) but did not meet the criteria for non-inferiority, meaning there was a potential for the device to be inferior to standard care. A recent patient-level meta-analysis found lower rates of hemorrhagic stroke with the Watchman device and non-inferior differences in the composite end point of all-cause stroke or systemic embolization. Of note, ischemic stroke per se occurred nearly twice as often in the Watchman arm compared to the warfarin arm (hazard ratio 1.95, p = 0.05) ^82^.

However, evidence also exists for improvements in quality of life ^83^, reduction in late bleeding ^84^, and economic benefit ^85^ with the use of LAA closure devices. More recently, other devices in addition to Watchman (although not yet FDA approved) have provided favorable outcomes ^86, 87^.

At present, we still do not know whether OAC-ineligible patients benefit from LAA closure, as the present trials were not inclusive of such patients, or how an LAA closure device would compare against the NOACs. Present treatment guidelines do state non-inferiority of LAA closure to standard-care warfarin, but this should be treated with caution as more data from long-term follow-up emerge.

## Conclusion

**Figure 1.**
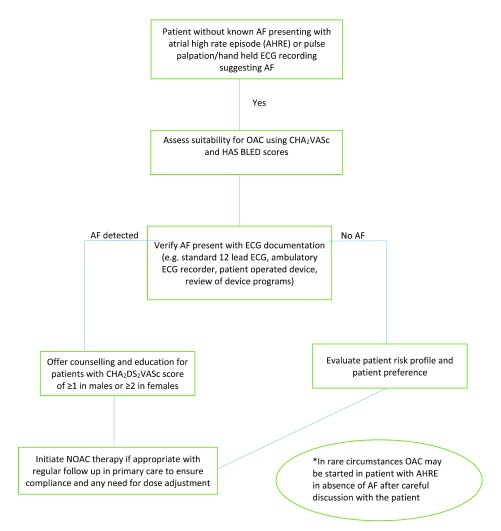
Proposed algorithm for the detection and management of atrial fibrillation (AF). CHA _2_DS _2_VASc, congestive heart failure, hypertension, age 65–74/>75, diabetes mellitus, stroke/transient ischemic attack/thromboembolism, vascular disease, female sex; ECG, electrocardiogram; HAS BLED, hypertension systolic blood pressure >160 mmHg, abnormal liver/renal function [with creatinine ≥200 μmol/L], stroke, bleeding history or predisposition, labile international normalized ratio [range <60% of the time], elderly [>65], concomitant drugs/alcohol; NOAC, non-vitamin K antagonist oral anticoagulant; OAC, oral anticoagulant.

The detection and management of AF is a core component of stroke prevention in the AF patient population. A proposed method of screening and managing AF is shown in Figure 1. With an increasingly aging population with multiple comorbidities, the diagnosis of AF becomes more likely. Most guidelines advocate simple opportunistic pulse check in primary care practices, but more prolonged forms of monitoring increase the yield of AF detection. With the introduction of NOACs, there appears to be little reason not to offer anticoagulation to all AF patients with one or more stroke risk factors, apart from those truly deemed “low risk” using the CHA _2_DS _2_VASc score.
